# Competitive growth mechanisms of AlN on Si (111) by MOVPE

**DOI:** 10.1038/srep06416

**Published:** 2014-09-18

**Authors:** Yuxia Feng, Hongyuan Wei, Shaoyan Yang, Zhen Chen, Lianshan Wang, Susu Kong, Guijuan Zhao, Xianglin Liu

**Affiliations:** 1 Key Laboratory of Semiconductor Materials Science, Institute of Semiconductors, Beijing Key Laboratory of Low Dimensional Semiconductor Materials and Devices, Chinese Academy of Sciences, P. O. Box 912, Beijing 100083, People's Republic of China; 2 LatticePower (Jiangxi) Corporation, No. 699 North Aixihu Road, National High-Tech Industrial Development Zone, Nanchang, Jiangxi, People's Republic of China, 330029

## Abstract

To improve the growth rate and crystal quality of AlN, the competitive growth mechanisms of AlN under different parameters were studied. The mass transport limited mechanism was competed with the gas-phase parasitic reaction and became dominated at low reactor pressure. The mechanism of strain relaxation at the AlN/Si interface was studied by transmission electron microscopy (TEM). Improved deposition rate in the mass-transport-limit region and increased adatom mobility were realized under extremely low reactor pressure.

III-nitride materials are extensively used in power electronic and optoelectronic devices. In particular, AlN film and its ternary alloys, which possess direct wide band gap as well as good thermal and chemical stability, extend the application of group-III nitrides to high temperature and high power field. Growth of group-III nitride materials on silicon has attracted much attention because of the potential integration with Si-based devices as well as the low cost and large size of Si substrate. However, due to the outdiffusion of Si, it is difficult to grow GaN or InN directly on Si substrate^1^. AlN has been widely applied as seed layer for GaN or InGaN grown on Si^2^,^3^,^4^,^5^,^6^. Moreover, the outstanding piezoelectric property and high acoustic velocity make AlN-on-Si an attractive approach for high frequency surface acoustic wave devices^7^.

To meet the needs of subsequent high quality GaN film growth and related devices fabrication, high quality AlN epitaxial layer is necessary. However, it is a challenging work to grow AlN film on Si because of the large lattice mismatch. And the gas-phase parasitic reaction in the process of metal organic vapor phase epitaxy (MOVPE), which is a successful method of depositing AlN material, deteriorates the quality of AlN^8^,^9^,^10^. Furthermore, insufficient mobility of AlN species on Si surface inhibits the structure rearrangement^11^. In addition, a main hindrance to the development of AlN film is the low growth rate. The reported growth rate of AlN layer on Si by MOVPE is rather low, for instance, 150 nm/h or ~160 nm/h^2^,^12^. The thickness of AlN seed layer used as template for subsequent GaN or InGaN growth generally needs to be 200–300 nm^4^,^5^,^12^,^13^,^14^. The growth inefficiency is the direct outcome of parasitic reaction of trimethylaluminium (TMAl) and ammonia (NH_3_) in vapor phase^15^,^16^,^17^,^18^. A kinetic model depicts that the gas reaction starts with the adduct formation from TMAl and NH_3_, then ends with nucleation and growth of AlN nanoparticles. Thermophoresis keeps the nanoparticles from reaching the substrate surface and results in precursor depletion^15^.

In this paper, the effects of the variation of basic epitaxial parameters, including growth pressure, flux of TMAl and NH_3_, on the growth rate and crystal quality of AlN on Si substrate are studied. The dependence of growth rate and crystal quality on precursor concentration under a quite low pressure is reported. The parasitic reaction and atom kinetics are taken into consideration to account for the competitive growth mechanisms.

## Discussion

**Figure 1:**
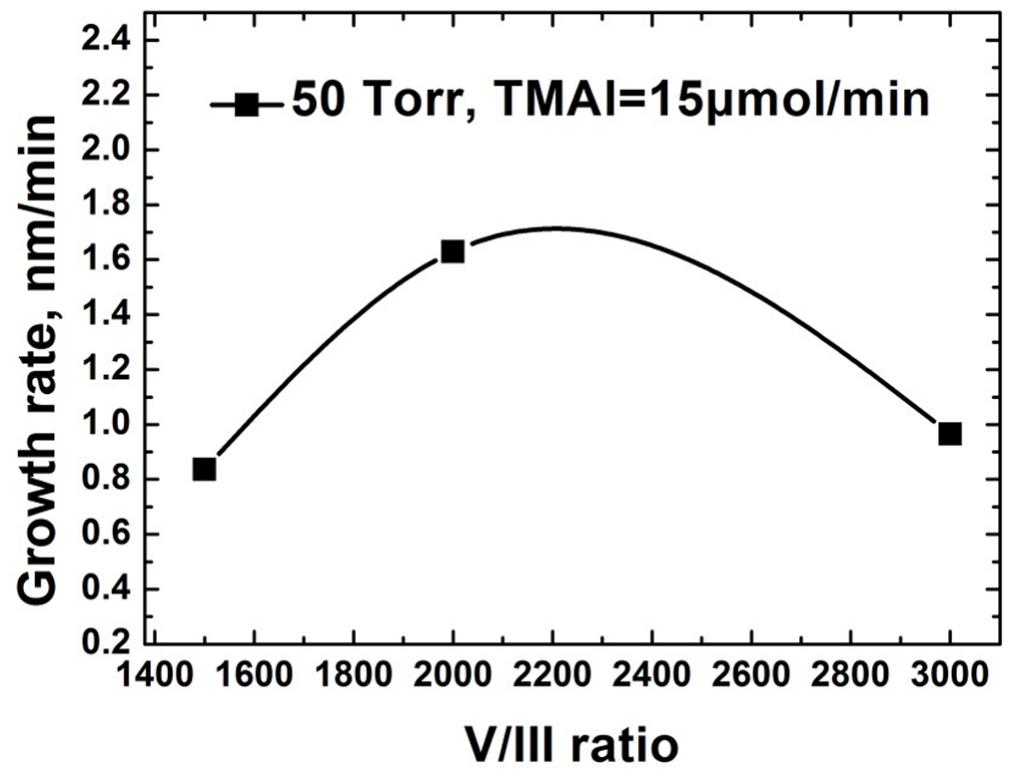
AlN growth rate versus V/III ratio at reactor pressure of 50 Torr.

**Figure 2:**
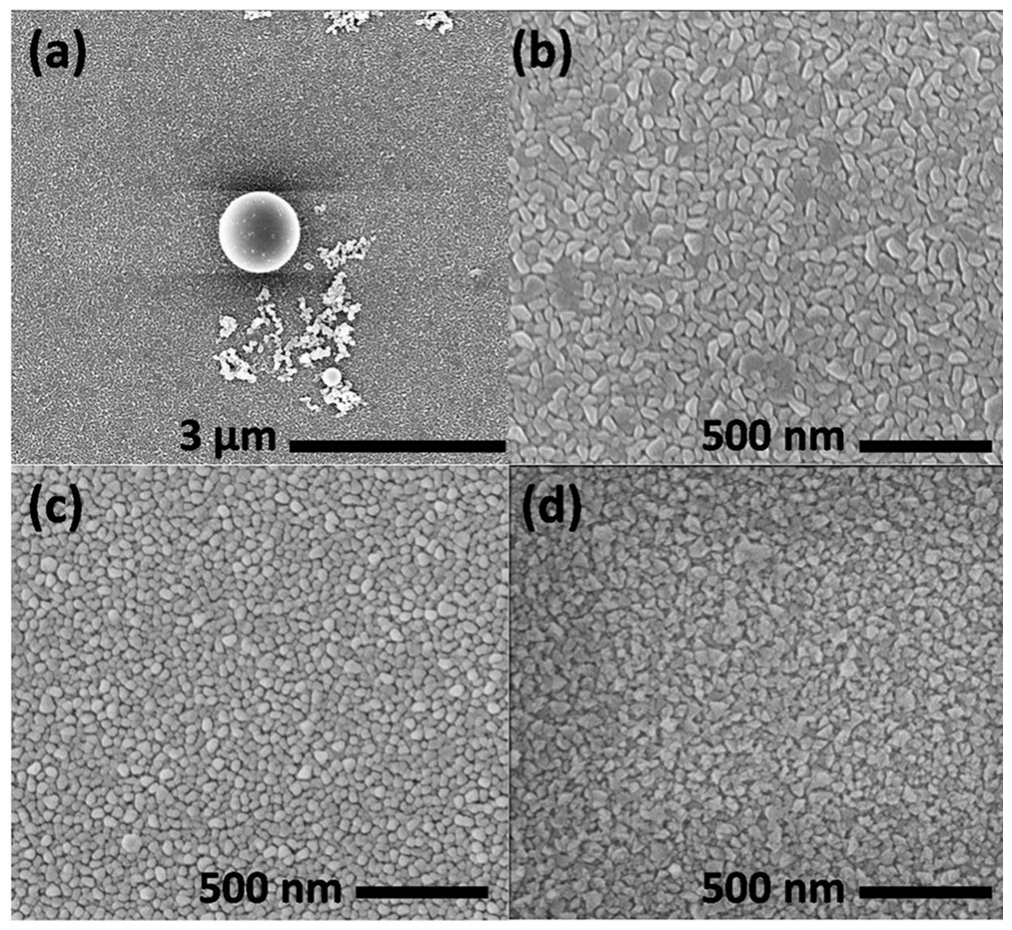
SEM images of AlN layers grown on Si (111) for 60 min at V/III ratio of (a), (b) 1500; (c) 2000; (d) 3000.

The change of AlN growth rate versus the V/III ratio under the reactor pressure of 50 Torr is shown in Fig. 1. The TMAl flux was fixed as 15 μmol/min and the total flow rate was kept constant, whereas the hydrogen/ammonia ratio was changed. The gas precursors had a residence time in MOVPE process, which involved complex gas phase and surface reactions. The low AlN growth rate was due to parasitic processes in the gas phase, which depleted the precursors through particles formation. The presence of white particles on the reactor sidewalls confirmed the severe parasitic reactions between group-III and group-V sources. Excess NH_3_ reduced the potential energy of Al(CH_3_)_3_·NH_3_ to form AlN particles^19^. As reported elsewhere, the AlN growth rate increased as the V/III ratio decreased from 3000 to 2000^20^,^21^. However, further reduction of NH_3_ flux led to decline of the AlN growth rate. It was attributed to the stoichiometric deteriorations. As shown in Fig. 2 (a), large Al droplets formed on the AlN surface at V/III ratio of 1500. The composition of droplet was determined by EDXS. Fig. 2 gives the surface morphology of AlN grown under the reactor pressure of 50 Torr for 60 min. The islands grown on Si (111) substrate were along c-axis, which determined by XRD (not shown here). The surfaces of samples grown at different V/III ratio appeared as island structure. Although decreased N can reduce group-III diffusion barrier energy^22^, there was little change of surface morphology with V/III ratio under these growth conditions. The insufficient mobility of adatoms on surface accounted for the rough island surface.

**Figure 3:**
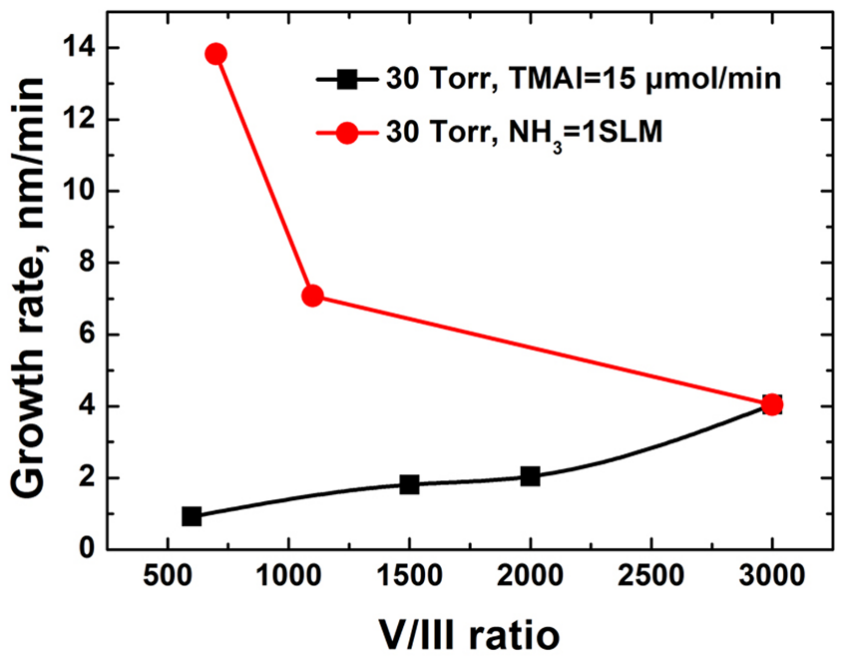
AlN growth rate versus V/III ratio at reactor pressure of 30 Torr.

**Figure 4:**
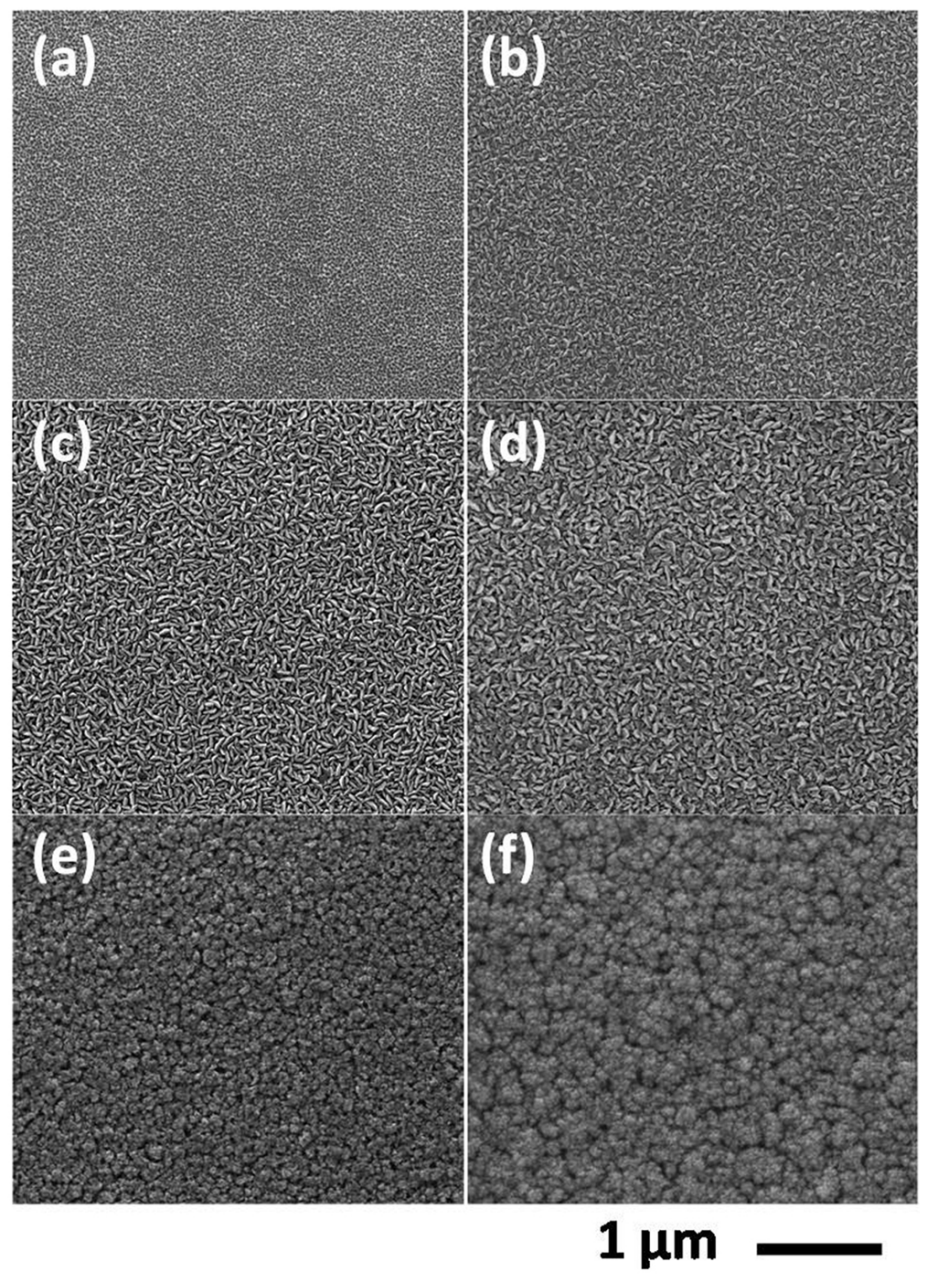
SEM images of AlN layers grown on Si (111) for 60 min at fixed TMAl flux of 15 μmol/min with V/III ratio of (a) 600, (b) 1500, (c) 2000, (d) 3000; at fixed NH_3_ flux of 1 SLM with V/III ratio of (e) 1100, (f) 700.

An effective way to minimize parasitic reaction is lowering the reactor pressure. The concentration of both reactants was reduced as the reactor pressure decreased. Meanwhile, a reduced residence time under low reactor pressure attenuated the parasitic reaction rate of AlN nanoparticle formation. Fig. 3 gives AlN growth rate variation with different V/III ratio under 30 Torr. The growth rate increases with the rising of NH_3_ flux at a fixed flux of TMAl being 15 μmol/min. It also increased with the increment of TMAl flux at a fixed flux of NH_3_ being 1 SLM. The dependence of growth rate on the flow rate of precursors was a clear indication that mass transport process limited the growth rate. The increased AlN growth rate during the mass transport limited growth suggested reduced parasitic reactions. The input partial pressure of group V source was much higher than that of group III. Therefore, the grow rate was more dependent on the TMAl flow rate than NH_3_, as shown in Fig. 3. Fig. 4 shows the SEM images of AlN samples grown for 60 min under 30 Torr. Fig. 4 (a) – (d) give the surface morphologies of AlN grown with increasing NH_3_ flux at a fixed flux of TMAl being 15 μmol/min. Fig. 4 (a) depicts porous structure while others show rice-like grain structure with different flux rate of NH_3_. As given in Fig. 4 (e) and (f), AlN grown at higher TMAl supply consisted of clusters of islands. AlN grown at a low NH_3_ flow rate of 0.2 SLM exhibited c-axis orientation, while other samples demonstrated randomly oriented AlN grains which exhibiting both (002) and (101) reflections (the XRD patterns are not shown here). Adatom diffusion was considered to be responsible for the material quality and surface morphology. For mass transport on the surface, the surface diffusion length *L_s_* was expressed as 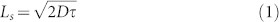
with *D* and *τ* being the diffusion coefficient and life time of Al adatom between the adsorption and desorption process. Diffusion coefficient was the measure of species mobility. According to Rosenberger^23^, the dependence of diffusion coefficient, *D*, on pressure and temperature was given by the semiempirical expression, 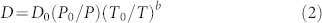
where *b, D_0_, P_0_* and *T_0_* were constants, *P* was the reactor pressure and *T* was the growth temperature. The reduced reactor pressure enhanced the mobility of adatoms on the surface. However, higher deposition rate at lower reactor pressure reduced the mean residence time of adatoms on the surface. Thus, a larger diffusion length could not be achieved under 30 Torr with increased diffusion coefficient and decreased mean residence time of Al adatoms. The three-dimension growth mode appeared with rough surface morphology and misoriented grains.

A large diffusion length, which should be larger than the terrace width, was essential to achieve the step-flow growth mode. As given by equation (1), the key factor to improve the crystal quality of AlN under high deposition rate is dramatically increasing the mobility of diffusing species. The diffusion coefficient could be increased by reducing the reactor pressure and rising growth temperature. However, increasing the temperature was usually restricted by system limitations. Reducing the reactor pressure was an effective way to enhance the mobility of adatoms. Meanwhile, it minimized the gas-phase parasitic reactions.

**Figure 5:**
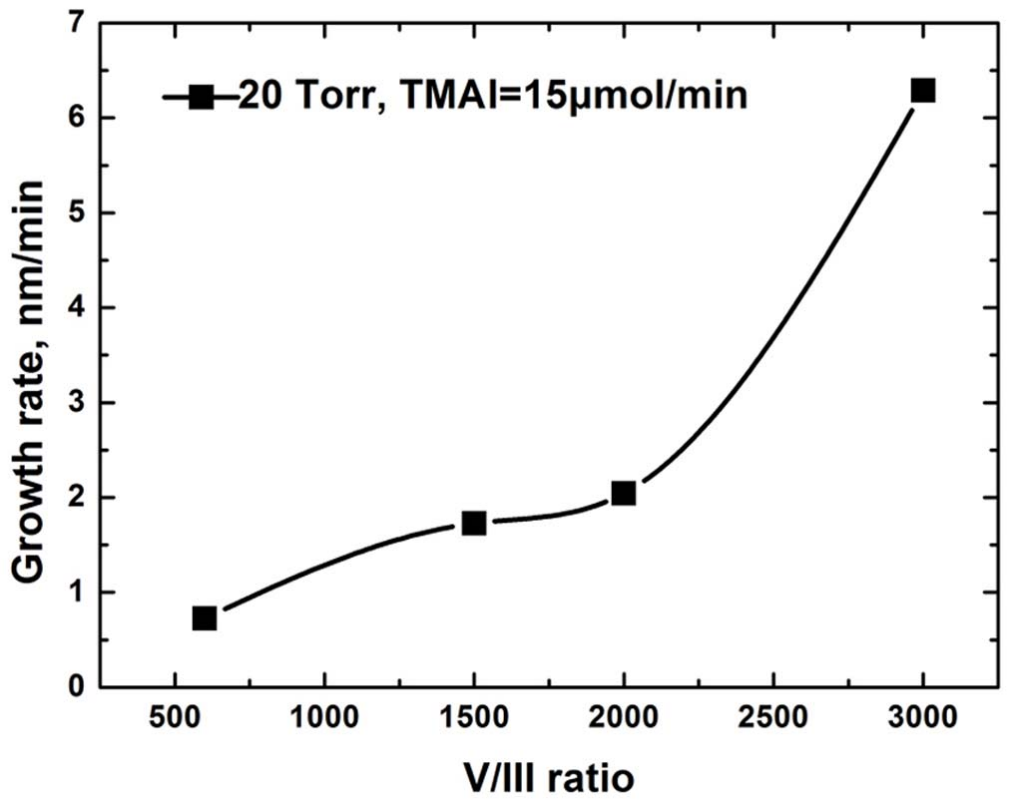
AlN growth rate versus V/III ratio at reactor pressure of 20 Torr.

**Figure 6:**
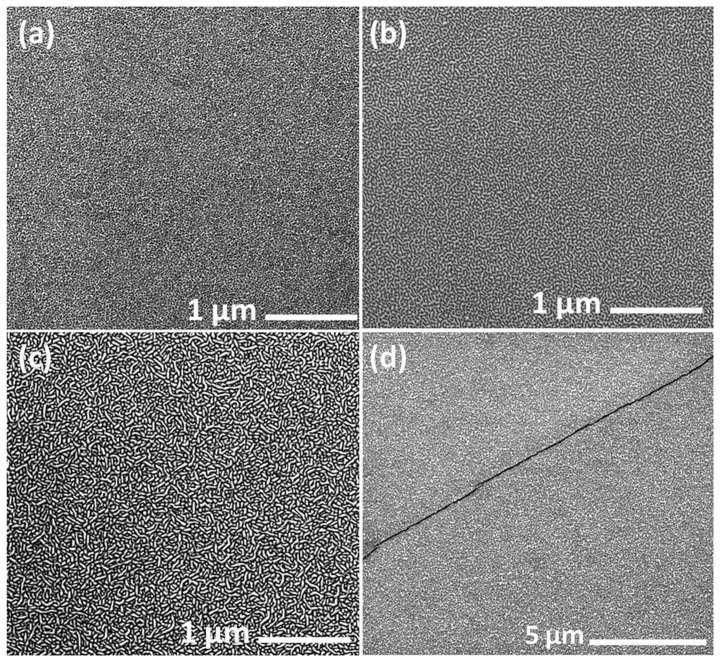
SEM images of AlN layers grown on Si (111) for 60 min at V/III ratio of (a) 600, (b) 1500; (c) 2000; (d) 3000.

As shown in Fig. 5, AlN growth rate increased as V/III ratio rose from 600 to 3000 under 20 Torr. The total flow rate was kept constant in the whole growth process. There was a significant increase in AlN deposition rate when the flux of NH_3_ rose to 1 SLM. The strong dependence of growth rate on the NH_3_ flux indicated that the mass transport limited mechanism was dominated rather than the parasitic reaction. This could be attributed to the lower gas-phase concentration of reactants and the resulting lower surface concentration of reactants at lowering pressure. The surface morphologies of AlN films grown under different V/III ratio are given in Fig. 6. All samples were deposited for 60 min. Although the thicknesses of AlN layers were different, the surface of the four samples showed resembling features with small pits or undulations. As observed by SEM in a small scale, AlN films grown at V/III ratio of 1500, 2000 and 3000 are heavily cracked because of the large thermal mismatch between AlN film and Si substrate. All of the four samples are well (002)-oriented as determined by XRD. The quasi-two-dimensional growth of AlN film indicated that reduced reactor pressure increased the surface diffusion length of deposited species. Growth rate as high as 380 nm/h of AlN was realized at the V/III ratio of 3000 under 20 Torr. The increased surface diffusion length and AlN deposition rate confirmed the enhanced lateral movement of adatoms at lowering reactor pressure, as expressed in equation (2).

**Figure 7:**
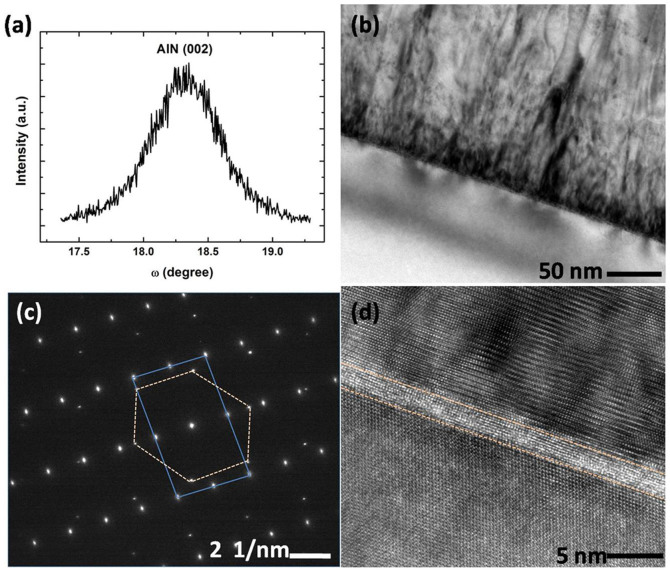
(a) DCXRD rocking curve of AlN (002) reflection; (b) cross-sectional TEM image; (c) SAED image; (d) HRTEM image of 380-nm-thick AlN grown under the reactor pressure of 20 Torr at V/III ratio of 3000.

To investigate the growth mechanism, AlN film grown at V/III ratio of 3000 under 20 Torr was studied by XRD and TEM. At first, the crystal quality of a 380-nm-thick AlN film was determined by DCXRD as shown in Fig. 7 (a). The x-ray rocking curve full-width at half-maximum (FWHM) for the AlN (002) reflection was 0.68°. The large linewidth value was mainly because there was no nucleation layer. Fig. 7 (b) depicts the cross-sectional TEM image of the 380-nm-thick AlN film grown at V/III ratio of 3000. Large numbers of dislocations generated at the interface and formed a highly distorted region with a thickness about 30 nm. The dislocations above the distorted region decreased as the layer thickness increased and the top layer was more perfect. HRTEM was applied to gain further insight into the interface between AlN film and Si substrate. An interfacial layer appeared at the position of 1.5 nm away from the AlN/Si interface. As confirmed by SAED in Fig. 7 (c), the interfacial layer consisted of hexagonal AlN rather than amorphous SiN_x_^24^. As shown in the HRTEM image, the abrupt character of the cubic/hexagonal lattice interface is an indication of coherent epitaxial growth. The large lattice mismatch was accommodated by the formation of misfit dislocations at the AlN (0001)/Si (111) interface with 4/5 matching^25^. The strain was released quickly within a couple of monolayers by the interfacial layer as above explained.

## Conclusions

The growth mechanisms of AlN under different conditions were investigated. The growth rate of AlN at normal pressure was limited by severe parasitic reaction. It decreased with the increase of NH_3_ flux. At lower growth pressure, the growth rate increased with rising of NH_3_ flux, which is a characteristic of mass transport limited process. Deposition rate of AlN was increased in the mass transport limited region because of mitigated parasitic reaction. Accompanied by improved adatom mobility, high quality AlN together with high growth rate can be obtained at extremely low pressure. The control of competitive growth mechanisms of AlN will be promising to contribute to the development of power electronics and optoelectronics.

## Methods

The AlN layer was grown on Si (111) substrate by a home-made metal organic vapor phase epitaxy (MOVPE) with a horizontal reactor. The group-III and group-V sources were transferred into the reactor separately in order to reduce the undesired parasitic reaction. TMAl and NH_3_ were employed as source of Al and N, respectively. The above-mentioned precursors were carried to the substrate surface by H_2_. After a cleaning step, Si substrate was loaded into the growth chamber to undergo a heat treatment at 1080°C in H_2_ atmosphere. AlN layers were grown on Si substrate after Al pre-deposition and no nucleation layer was grown before the deposition of AlN layer. The growth temperature was fixed at 1080°C, and other epitaxial parameters such as growth pressure, the flux of TMAl and NH_3_ were adjusted individually.

The morphologies of AlN layers were observed using field emission scanning electron microscopy (FE-SEM: Hitachi S-4800 and Quanta FEG 450). The composition of the AlN was examined by energy-dispersive X-ray spectroscopy (EDXS). The thickness of AlN layers were measured ex situ by both cross-section SEM and spectroscopic ellipsometry (M-2000DI) at 633 nm with an illumination spot diameter of 300 μm. Crystallographic characterization of AlN layers were carried out by XRD in ω/2θ scan mode (Rigaku-TTRiii). The crystallinity of AlN film was determined by double crystal X-ray diffraction (DCXRD) using CuK_α1_ (Philips X-pert) radiation source. The structure of AlN/Si was studied by conventional transmission electron microscopy (TEM: FEI G2 F20), high-resolution TEM (HRTEM) and selective area electron diffraction (SAED).

## Author Contributions

Y.F. contributed to the design experiments with assistance of S.Y., Z.C., L.W. and X.L., Y.F. grew the samples and wrote the manuscript under the help of H.W., S.K. and G.Z. All authors discussed the results and reviewed the manuscript.
